# Structure-based designing efficient peptides based on p53 binding site residues to disrupt p53-MDM2/X interaction

**DOI:** 10.1038/s41598-020-67510-8

**Published:** 2020-07-10

**Authors:** Nasim Rasafar, Abolfazl Barzegar, Elnaz Mehdizadeh Aghdam

**Affiliations:** 1 Research Institute of Bioscience and Biotechnology, University of Tabriz, Tabriz, Iran; 2 Molecular Medicine Research Center, Biomedicine Institute, Tabriz University of Medical Sciences, Tabriz, Iran; 3 Department of Pharmaceutical Biotechnology, Faculty of Pharmacy, Tabriz University of Medical Sciences, Tabriz, Iran

**Keywords:** Drug development, Computational models

## Abstract

MDM2 and MDMX are known as overexpressed oncoproteins in several wild-type p53 cancer cells. The development of potent and dual antagonist peptides for p53-MDM2/X is a continuous challenge. In this study, we intended to investigate the pivotal structural points respecting the development of potent and dual inhibitors of MDM2/X. Correspondingly, MD simulation was performed on the experimentally confirmed peptides, comprising p53, pDI, pDIQ, PMI, and computationally screened mutant pDI and pDIQ. A follow-up secondary structure analysis showed the last three C-terminal residues provide the helicity reservation of peptides bound to MDM2/X. Furthermore, a delicate residue-residue examination displayed Met 11 and Ser12 in the modified peptides contribute significantly to dual inhibition of MDM2/X. Additionally, the peptides_MDM2/X complexes’ ΔG_binding_ extracted by the umbrella sampling method were in agreement with the pattern of their experimental affinity values. It was concluded the screened pDI mutants were considered as suitable anti-MDM2/X peptides, and the data obtained could be exploited as the theoretical structure-based guide for rational peptide design. Taking account of results, the suitable C-terminal residues of p53-based peptides especially Met11, and Ser12, as well as higher umbrella sampling, generated ΔG_binding_ to MDM2/X would be considered as the positive structural markers of a promising anti-cancer agent.

## Introduction

The mutation of p53 protein is one of the frequent causes of human cancer since it has a key role in numerous cell stability and proliferation functions^1^. On the other hand, MDM2 is an oncogene protein that negatively regulates p53^2^. MDMX (MDM4) is also a homolog for MDM2 and acts as p53 inhibitor^3^. In vivo and in vitro studies have shown the overexpression of both MDM2 and MDMX proteins in several cancer types^4^ (e. g. MDMX overexpression up to 92% of AML cases^5^). However, their mechanisms of action are slightly different as MDM2 mainly degrades p53 protein using its E3 ubiquitin ligase activity, while MDMX suppresses p53 by decreasing its transactivation activity and increasing MDM2 function^6^.

Both MDM2 and MDMX (MDM2/X) are significantly overexpressed in cancer cells harboring wild type p53^7,8^. As a result, the inhibition of MDM2/X-p53 interaction leads to the restoration of p53 activity and subsequent tumor suppression^9^. From the structural point of view, MDM2/X share a similar p53-binding domain of the N-terminal hydrophobic pocket. This site engages hydrophobic bonds with p53 residues of Phe19, Trp23, and Leu26, which leads to the p53 transcriptional activity suppression^10,11^.

To pharmacologically antagonize the MDM2/X-p53 interaction, several molecules have been developed. In this path, small molecule inhibitors, such as Nutlin-3 were mostly unsuccessful in clinical trials^12^. Although its derivative, RG7112, has shown some promising results, its unsuitable inhibition of MDMX was one of the main drawbacks^13–15^. Alongside small molecules, some lead peptide sequences have also been developed, mostly by the alteration in the p53-MDM2/X binding site (ETFSDLWKLLPE), including 12/1 (MPRFMDYWEGLN)^16^, _ENREF_17PMI (TSFAEYWNLLSP)^17^ and pDI (LTFEHYWAQLTS)^18^. In what follows, there are several studies to improve the kinetics and dynamics of these peptides such as synthesizing stapled peptides^19,20^. One of the first examples was SAH-8, designed by stapling p53-MDM2/X binding site sequence^21^. More recently, ATSP-7041 has been developed as stapled pDI peptide which later led to ALRN-6924 peptide with promising anticancer results^22^. Nevertheless, the main limitation in this area is the common low affinity of peptides to MDMX protein which reduces their effectiveness as anticancer agents^23,24^. As a result, the optimization of lead peptides to obtain a “potent” and “dual” inhibitor of MDM2/X is an ongoing challenge. “Potent” is a common term for high affinity and stable therapeutic candidate which targets MDM2/X. On the other hand, any agent introduced to inhibit MDM2 is not robust enough to have an acceptable cytotoxic effect unless it could also inhibit MDMX.^18^. Consequently, “dual” refers to the capability of the peptides to target both MDM2 and MDMX proteins. Most of the previous studies focused on the rational design of p53-based peptides are usually lacking the consideration of dual inhibition of MDM2/X^25,26^. In this regard, one of the promising approaches is using in silico and theoretical workflows such as Molecular Dynamic (MD) simulation to optimize and verify drug candidate peptides^27–29^. Accordingly, in this study, we applied a computational structure-based approach inclusively on experimentally validated p53-based peptides, including p53, pDI, pDIQ, and pMI to; A) study their “potent” and “dual” inhibitor activities against MDM2/X, B) find critical residues playing roles in higher affinity to MDM2/X and helicity conservation, C) follow a computational high-throughput point mutation screening on pDI, (LTFEHYWAQLTS) and pDIQ, (ETFEHWWSQLLS) to analyze and validate structurally and functionally. To these ends, 27 different MD simulations and follow-up analyses were performed to assign the suitable features of native and mutated peptides against MDM2/X. The appropriate correlation between achieved theoretical and confirmed experimental results highlighted the suitability of the suggested methodology in this study, which could lead to the implicit rational peptide design in this area.

## Materials and methods

### Atomic coordinates of peptide-MDM2/X structures

The structures of peptides-MDM2/X complexes were extracted from Protein Data Bank (PDB). First, due to the lack of pDI-MDM2 structure with the total number of residues, the two close PDB codes of 3G03 and 3JZR (https://www.rcsb.org/) were taken. The final coordinates were achieved by the superimpositions and refinement of the mentioned PDB structures utilizing Chimera software. The coordinates of other structures were directly extracted from PDB codes of 3FDO, 4HFZ, 3DAB, 3EQS, 3EQY, 3JZS, and 3JZQ for pDI-MDMX, p53-MDM2, p53-MDMX, PMI-MDM2, PMI-MDMX, pDIQ-MDM2, and pDIQ-MDMX, respectively. The “repair PDB” command of FoldX v.3 program was employed to optimize the structures and add the missing atoms.

### High-throughput mutation screening of pDI and pDIQ using FoldX

To screen a large number of possible mutations of pDI and pDIQ peptides, the FoldX program was applied. The FoldX program computes the interaction energy robustly based on the empirical data extracted from protein engineering studies which makes it a notable assistance for peptide designing. First,_ENREF_29 the “PSSM” interface of FoldX was utilized to mutate all the residues of peptides to 20 native amino acids (mutation to the residue itself was also carried out) in pDI-MDM2/X and pDIQ-MDM2/X complexes. Using BuildModel and AnalyseComplex interfaces upon each mutation, the final optimized structure and the interaction energy of the peptide-MDM2/X complexes were obtained. The FoldX force field was used to calculate the free energy of unfolding (ΔG) as described below.

$$\Delta G = \left( { 0.33 \times \Delta G_{{{\text{vdw}}}} } \right) + \Delta G_{{{\text{solvH}}}} + \Delta G_{{{\text{solvP}}}} + \Delta G_{{{\text{wb}}}} + \Delta G_{{{\text{hbond}}}} + \Delta G_{{{\text{el}}}} + \Delta G_{{{\text{kon}}}} + T\Delta S_{{{\text{mc}}}} + T\Delta S_{{{\text{sc}}}}$$

where the terms are free energy difference of total van der Waals energy of all atoms (Δ*G*_vdw_); solvation energy for hydrophobic and polar groups (Δ*G*_solvH_ and Δ*G*_solvP_ , respectively); the water bridges (Δ*G*_wb_); the hydrogen bonds (Δ*G*_hbond_); the electrostatic bonds (Δ*G*_el_ and the electrostatic bonds of subunit molecules (Δ*G*_kon_). Meanwhile, Δ*S*_mc_ is defined as the entropy change when the backbone is changed to a particular conformation, and Δ*S*_sc_ is the entropy change when the side chain is transformed into an appropriate structure. *T* is the temperature^30^.

The input structures of pDI-MDM2/X and pDIQ-MDM2/X for the FoldX program were described in the “Atomic coordinates of peptide-MDM2/X structures” section. After each mutation on the peptides’ residues, the variance of interaction energy (ΔE) was calculated. Subsequently, the extracted structures with the lowest ΔE were selected for further studies. The final atomic coordinates of selected mutant peptides_MDM2/X complexes were used as reference structures for MD simulations.

### Molecular dynamics simulations

GROMACS-2018 package^31^ was applied to perform 200 ns simulation on 27 different systems (total time of 5.4 μs) using the GROMOS96 54a7 force field. Solvation of each system was conducted via the SPC water model in the a triclinic box that the distances of molecules’ centers to the edges were 15 Å. All the simulation systems were neutralized by the suitable number of Na + and Cl- ions with the final ionic concentration of 150 mM. The van der Waals interactions were truncated using energy and pressure correction of long-range dispersion. Particle Mesh Ewald (PME) technique and LINCS algorithm^32^ were employed for the calculation of long-range electrostatic interactions and making all bond constraints, respectively. Furthermore, the integration time step was set up as 2 fs and periodic boundary conditions were utilized in all directions. Prior to MD simulation, 200 ps of energy minimization from 0 to 300 K was carried out using a method of steepest descent. Moreover, 200 ps of system equilibration at 300 K was conducted with constant volume (NVT) and constant pressure (NPT) ensembles. UCSF Chimera Viewers was used to visualize the simulation outcomes. In addition, the analysis of Dictionary of Secondary Structure for Proteins (DSSP) of all complexes was conducted during the simulation time. Follow-up “umbrella sampling” and “gRINN” analyses were also conducted as explained in the following sections.

### Umbrella sampling

Umbrella sampling simulation has proven to be a robust method to obtain the ΔG of a protein–protein interaction using the extracted potential of mean force (PMF)^33^. In this approach, after the converging of PMF curve at a large center of mass distance, the difference of PMF’s highest and lowest values is calculated as ΔG. The center of mass is defined as the representing average point of the mass. Moreover, the “wham utility” of the GROMACS package is used to perform the Weighted Histogram Analysis Method (WHAM) to extract the values of PMF during the simulations.

The whole procedure is described as follows. For pulling simulations, the atomic coordinates resulted from the last trajectories of MD simulations, as described in the “Molecular dynamics simulations” section, were used as starting structures. Prior to pulling simulation, energy minimization and the only 200 ps of NPT ensemble were conducted. Subsequently, immobile references were adjusted for pulling simulations via the induction of restraints on MDM2/X proteins. The pull rate of 0.001 nm ps^−1^ (0.01 Å ps^−1^) and the spring constant of 1,000 kJ mol^−1^ nm^−2^ were used in the pulling of the peptides outside the structures’ centers along the corresponding axis over 2.5 ns. The final COM distance between peptides and MDM2/X proteins was approximately 5 nm. In the next step, an approximate number of 25 snapshots of pulling simulations’ trajectories were taken to obtain the primary structures for the windows of umbrella sampling. Moreover, to have an asymmetric distribution of sampling windows, the window spacing was defined as 0.1 nm up to 0.2 nm, beyond 3 nm of COM separation. Afterward, each window was utilized for 5 ns of MD simulation which makes the total umbrella sampling simulation time of approximate 1,125 ns. Ultimately, the PMF curve was produced and the differences of the maximum and minimum values of PMF were used to obtain the ΔG_binding_ for different complexes of peptides_MDM2/X by WHAM utility analyses.

### Analyses of residue interactions in peptide-MDM2/X complexes using gRINN tool

One of the significant outputs of MD simulations is the functioning behavior of each residue especially in the protein–protein interactions. As a result, to gain information about the peptide_MDM2/X interactions on the residue level, a post-simulation analysis, called gRINN (get Residue Interaction eNergies and Networks) v1.1.0.hf1 tool, was utilized^34^. This software uses MD simulation trajectories for building any pairwise amino acids non-bonded interaction energies. Before introducing the files into the software, all water and non-protein molecules were removed. The outputs of MD simulations including “tpr”, “top” and “xtc” files were used as input files for gRINN tool. The generated data from 2000 MD simulation trajectories were employed to extract important residues interaction energy based analysis.

## Results and discussion

### Mutant pDI and pDIQ peptides screening in the complex with MDM2/X

MDM2 and MDMX are considered as well-known oncoproteins with inhibitory activities on the p53 protein. In addition, they are overexpressed in several types of cancerous cells harboring wild type p53^4^. Therefore, MDM2/X suppression strategies have been considered as promising approaches for cancer therapy over the last decade^7,9^. In this path, anticancer peptides are the robust agents to inhibit MDM2/X proteins effectively^17,18,21–23^. Hence, we have studied some p53-based peptides computationally to obtain high affinity, stable, and valid anti-MDM2/X peptides. In addition, we aimed to qualify our theoretical methodology for the development of “dual” anti-MDM/X peptides. To this end, a lead peptide named pDI^18^ and its more potent derivative, pDIQ^17^ were used as starting peptides for the mutation screening using the FoldX program.

Having used the PSSM interface of the FoldX program, the mutations were scored based on their primary interaction energy (∆E) of the mutated pDI and pDIQ peptides in complex with MDM2/X. The corresponding results were displayed in Tables S1, S2, S3, and S4. The criterion to select the mutant peptides was the appropriate ∆E for both MDM2/X to propose a dual inhibiting peptide. Excluding the neutral positions showing ineffective ∆E values, some point mutations on pDI with suitable interaction energies with MDM2/X were selected (Tables S1 and S2). The same procedure was performed on pDIQ_MDM2/X complexes (Tables S3 and S4). In summary, Leu1, His5, Ala8, Gln9, Ser12 were considered as neutral positions in pDI_MDM2 complexes as their ∆E showed neither negative nor positive value. Thr2 was also a neutral position to mutate in the pDI_MDMX complex. However, mutations on Phe3, Trp7, Leu10 residues were shown to be non-favorable. This finding is consistent with the key residues of Phe19, Trp23, and Leu26 as p53 binding segment with MDM2/X, mentioned in other studies^11^. Conversely, single mutations on Glu4 and Thr11 were identified with favorable ∆Es in both MDM2/X complexes. Moreover, double mutations on the same positions of pDI were carried out and ∆Es were obtained. Accordingly, three pDI mutants, pDI_E4W (pDIm1) (∆E = −1.97 and − 2.89 kcal/mol), pDI_T11M (pDIm2) (∆E = −1.58 and − 2.02 kcal/mol) and the double mutant pDI_E4WT11M (pDIdm) (∆E = −1.40 and − 1.01 kcal/mol) were selected with the most suitable ΔEs for MDM2 and MDMX, respectively (Tables S1 and S2). Regarding pDIQ, single mutations in Thr2 and Glu4 were shown to indicate favorable ∆Es in both MDM2/X complexes. As a result, the best mutant candidates of pDIQ were pDIQ_E4K (pDIQm1) (∆E = −1.26 and − 0.83 kcal/mol) and pDIQ_T2H (pDIQm2) (∆E = −1.28 and − 0.90 kcal/mol) with the best ΔEs for MDM2 and MDMX, respectively (Tables S3^3^and S4). The extracted atom coordinates from the mentioned mutants, as well as the p53, pDI, pDIQ, and PMI peptides were collected for further MD simulations in the free and MDM2/X bound states.

### Secondary structural stability of peptides in the free state and complex with MDM2/X

The different experimentally validated anti-MDM2/X peptides, including p53, pMI, pDI, pDIQ, and also the selected mutant peptides (see the “Mutant pDI and pDIQ peptides screening in the complex with MDM2/X” section) were entered into the MD simulations in the free and complex states. The sequences and available experimental IC_50_ of each peptide are shown in Table 1.

**Table 1:** IC_50_ and computational ΔG_binding_ of the peptides_MDM2/X. The experimental IC_50_s recorded in the literature ^17^ for control studied peptides of p53, pDI, pDIQ, and PMI are presented. Also, ΔG_binding_s of all studied peptides of p53, pDI, pDIm1, pDIm2, pDIdm, pDIQ, pDIQm1, pDIQm2 and PMI in complex with MDM2/X using the “umbrella sampling” method are shown.

Peptide	Sequence	IC_50_ (nM)		ΔG_binding_^a^ (kCal/mol)	
		MDM2	MDMX	MDM2	MDMX
p53	ETFSDLWKLLPE	2000	6000	21	NA
pDI	LTFEHYWAQLTS	44	550	22	20
pDIm1	LTFWHYWAQLTS	–	–	28	24
pDIm2	LTFEHYWAQLMS	–	–	28	22
pDIdm	LTFWHYWAQLMS	–	–	25	28
pDIQ	ETFEHWWSQLLS	8	110	31	16
pDIQm1	EHFEHWWSQLLS	–	–	25	19
pDIQm2	ETFKHWWSQLLS	–	–	18	13
PMI	TSFAEYWNLLSP	20	40	25	19

^a^The error with the calculated energy is 0.2 kcal/mol for every system.

NA is not applicable.

**Figure 1:**
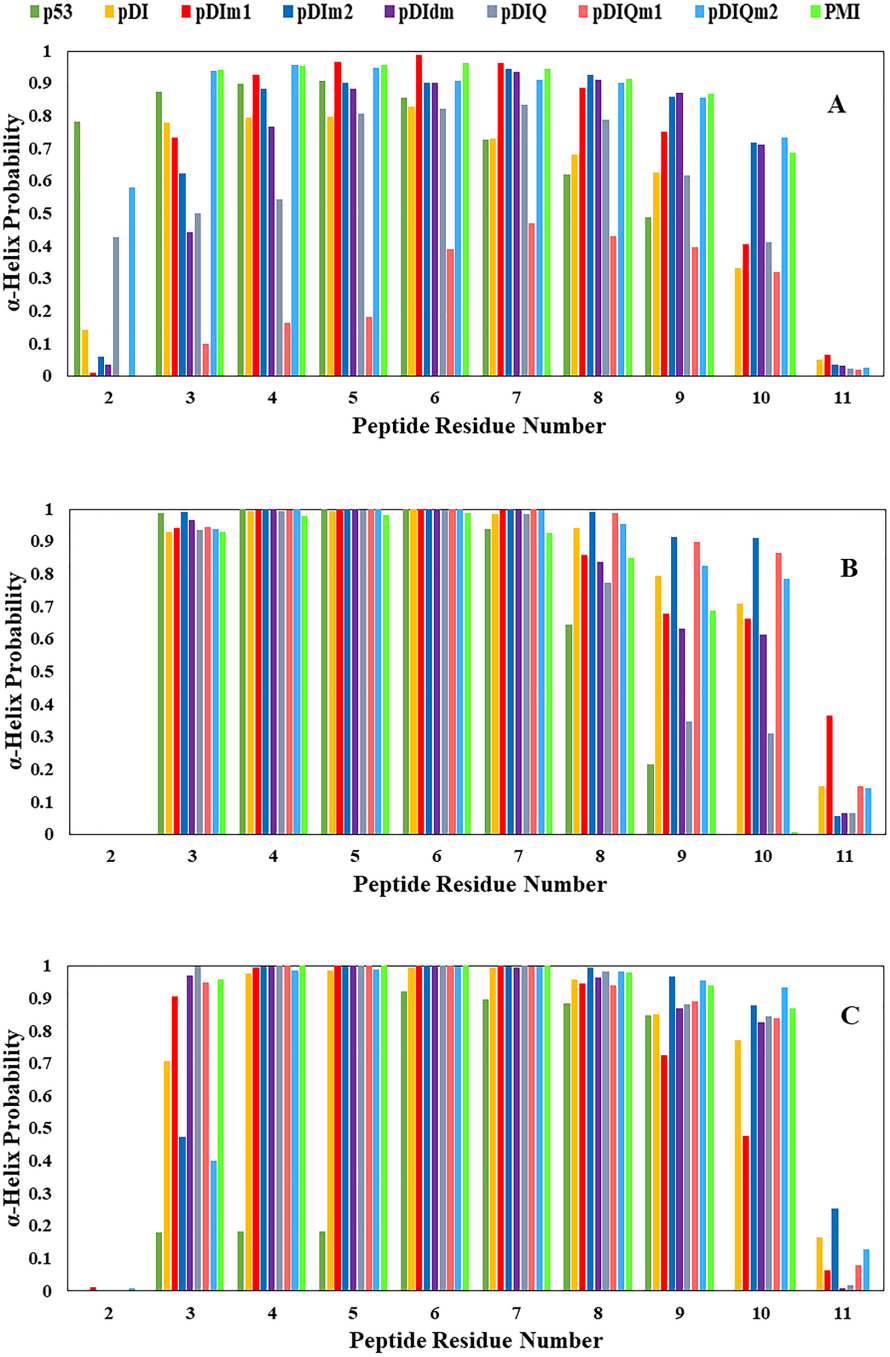
Helicity probability of the studied peptides during MD simulation of different studied peptides of p53, pDI, pDIm1, pDIm2, pDIdm, pDIQ, pDIQm1, pDIQm2 and PMI using DSSP program in the free state (**A**), in complex with MDM2 (**B**) and in complex with MDMX (**C**).

Peptide-based agents, especially with the resemblance to the p53-MDM2/X binding site, have been shown promising dual inhibition of MDM2/X. The mentioned binding site consists of 12 residues with an alpha-helix structure. Helical peptides are usually unstable without a complete protein fold which reduces their affinity to the target protein. It is therefore highly significant to preserve the helicity of such peptides. In this regard, secondary structure analysis was performed on the studied peptides both in the free state and in complex with MDM2/X, using DSSP (Dictionary of Secondary Structure of Proteins) program^35^. The results are depicted in Fig. 1.

As shown in Fig. 1A, the free p53 peptide interestingly showed one of the best conformational stability in the N-terminal residues (Thr2 and Phe3) compared to the same positions in other peptides. Likewise, in the pDIQm2 and PMI peptides, phe3, one of the conserved residues in the p53 binding site, preserved helicity.

The pDIQm1 peptide has the lowest helicity scores among all of the free mutant peptides. Regarding C-terminal residues, in the free p53 peptide, Leu9 has a score of 0.48, and Leu10 and Pro11 showed no helicity. In fact, Proline is known for destabilizing alpha-helix structures especially in the C-terminal residues^36^ and this is one of the reasons p53 lost its helicity during the 200 ns simulation. However, other free peptides showed diverse range of stability in alpha-helix conformation in residues 8 to 11 (Fig. 1A). Overall, the mutant pDI and pDIQ peptides showed the best helicity in all Ala8, Gln9, Leu10, and Met11 compared to other studied free peptides. In the complex forms with MDM2/X, as expected, the conformational stability of most residues was increased (Fig. 1B, C). However, in contrast to the free states, residue 2 of the peptides in complex with MDM2/X showed no or very little score of helicity (Fig. 1B, C). Moreover, in residue 3, the helicity scores were less and were varied in the peptides_MDM2/X complexes (Fig. 1B, C). Residue 11 of PMI bound to MDM2 showed no helicity compared to its free state (Fig. 1B). In the p53_MDMX, peptide residues indicated lower helicity scores in the positions 3 to 5 rather than the scores of the same positions in the p53_MDM2. The conformational stability of pDIQm2 was also increased in complex forms possibly due to the favorable interaction energy between the peptide and MDM2/X. The overall results confirmed the improvement of the conformational stability of the previously designed peptides (pDI, pDIQ, and PMI) both in the free and complex forms compared to p53. Moreover, the newly developed mutant peptides especially pDIm2, pDIdm, and pDIQm1 have shown the best helicity score, particularly in the C-terminal residues. The importance of the C-terminal of p53-based peptides has previously been shown in the binding features to MDM2/X^37,38^. Specifically, Pazgier et al*.* have shown that p53 peptides C-termini mutation from proline to other amino acids lead to an extended alpha-helical conformation in complex with MDM2^17^. Overall, data in Fig. 1 clearly displayed that; A) almost all peptides preserved their initial helicity structure during 200 ns simulation in the free and mainly in the complex forms. B) the helicity values of peptides in complex with MDM2 were more dominant than those of MDMX. C) The helicity probability had almost a similar pattern in residues 3 to 10 in the free state. However, the helicity score was higher in N-terminal residues rather than C-terminal residues in the complex states (mainly with MDM2).

It should be noted that the first two and the last residues did not participate in helix structure, neither in the free nor the complex states. However, their effect on the helicity extension especially on C-terminal residues was undeniable. The impact of the last residue on the peptides’ structures will be discussed in the following sections.

### Binding free energy analysis of peptides_MDM2/X using the umbrella sampling method

**Figure 2:**
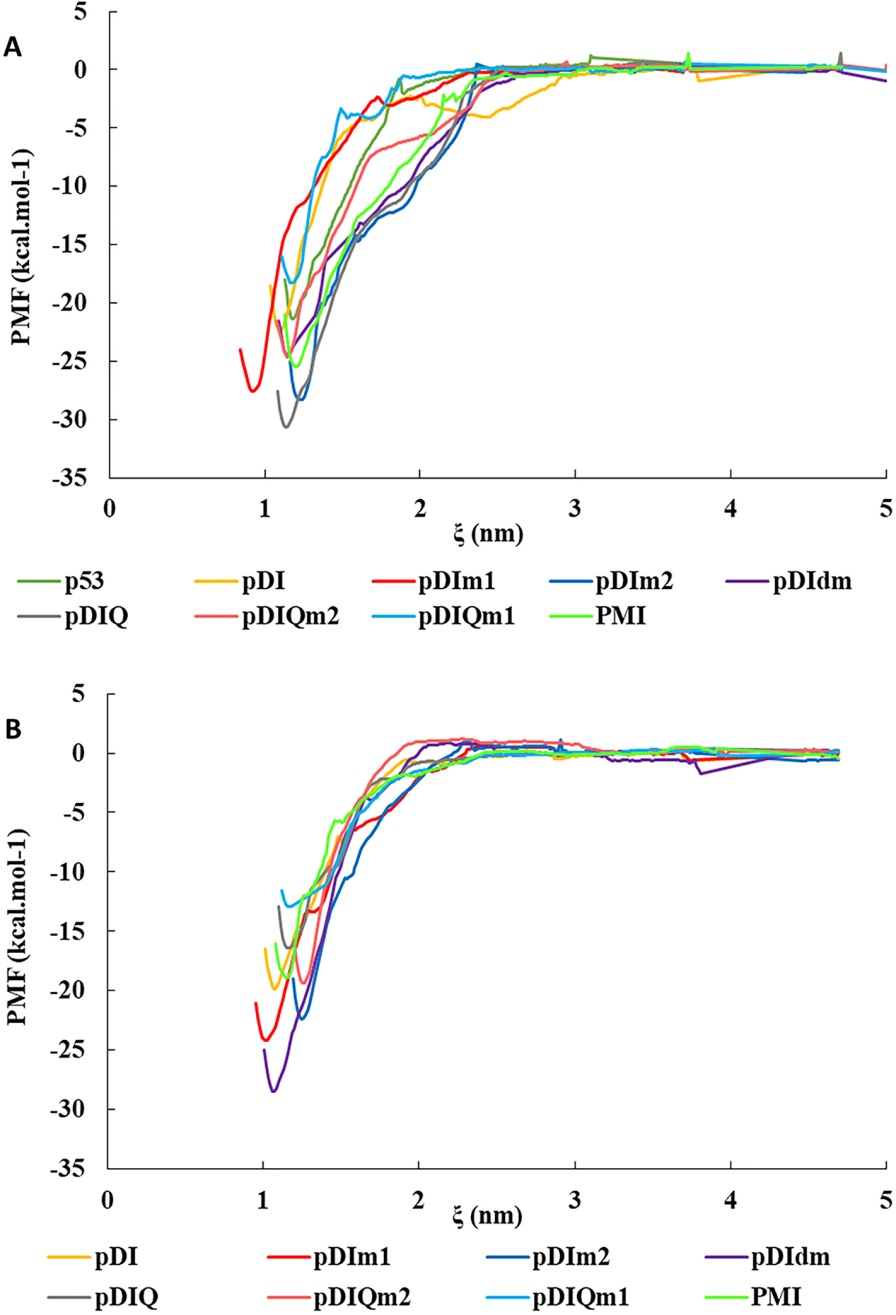
Potential of mean force (PMF) curve compared to the mass center between studied peptides and (**A**) MDM2 and (**B**) MDMX using the “umbrella sampling” method. ξ is the center of mass between peptides and MDM2/X in angstrom.

The binding affinity of the therapeutic agents to the target proteins is an important factor in predicting their effectiveness and value as promising drug candidates. It is therefore essential to precisely calculate the ΔG_binding_ of such interactions. The umbrella sampling method is a trustworthy approach for ΔG_binding_ computation especially for protein–protein interactions^33,39^. Hence, in the current study, the interaction energies of all peptides-MDM2/X complexes were calculated using the umbrella sampling method. The method uses the PMF profile of a pulling simulation to estimate the binding energy between the two molecules. According to Fig. 2 and Table 1, the ΔG_binding_ of pDI mutant peptides to MDM2/X remarkably increased compared to the control peptides mainly p53 and pDI. The ΔG_binding_ results of pDIm1 and pDIm2 were also more suitable compared to the PMI peptide in complex with MDM2/X. The pDIQ peptide as an experimentally valid and potent inhibitory agent with previously measured IC_50_ of 8 and 110 nM^17^ showed 31 and 16 kcal/mole ΔG_binding_ for MDM2 and MDMX, respectively (Table 1). These results were highly consistent with the experimental results which were also plausible for p53, pDI, and PMI peptides. As shown in Table 1, the order of peptides affinity to MDM2 is pDIQ > PMI > pDI > p53 based on the experimental results. Interestingly, the computational calculation showed the same order. This was also applicable to MDMX complexes with a slight tolerance. As a result, the suitable binding energies of pDIm1, pDIm2, and pDIdm peptides with MDM2/X could acquire more validity. On the other hand, the pDIQm2 peptide had less binding energy to MDM2 and more affinity to MDMX compared to pDIQ itself. In addition, its binding energy was equal to PMI’s. However, the pDIQm1 peptide did not have a suitable affinity to MDM2/X as the pDIQ peptide had. Given the fact that pDIQ is an optimized sequence, it seems more mutations such as E4K could not effectively improve the affinity to MDM2/X proteins.

### Residual interaction analyses of peptides_MDM2/X using gRINN method

**Figure 3:**
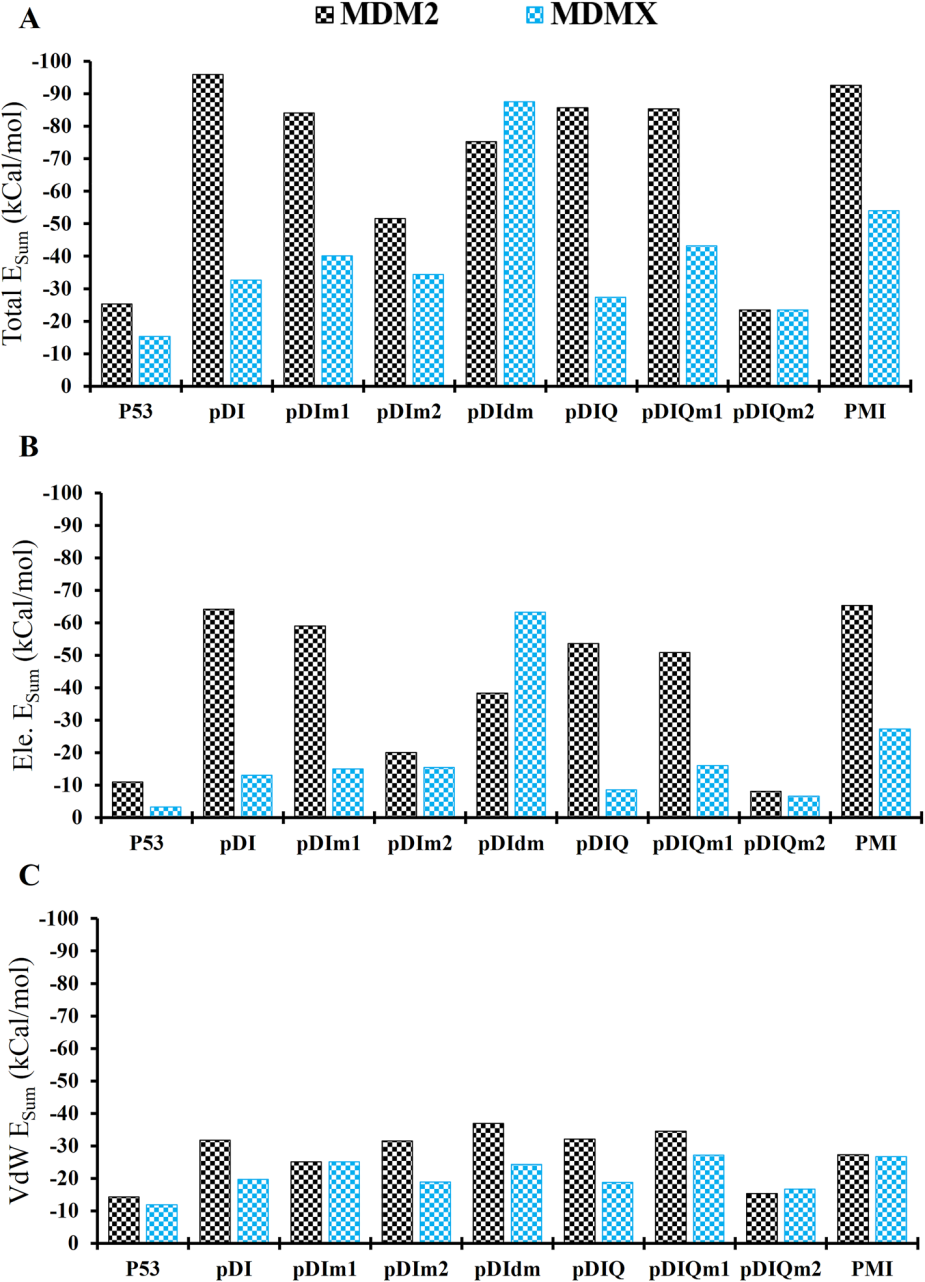
Sum of interaction energies between studied peptides of p53, pDI, pDIm1, pDIm2, pDIdm, pDIQ, pDIQm1, pDIQm2, and PMI in complex with MDM2 and MDMX during MD simulation generated by gRINN tool. Black and Blue columns are MDM2 and MDMX complex forms, respectively. (**A**) illustrates the sum of all types of interaction energies, (**B**) indicates the total sum of electrostatic type of interaction energies, and (**C**) shows the total sum of van der Waals type of interaction energies in peptides_MDM2/X.

Each residue of a peptide can stimulate a dramatic structural and functional change in the peptide_protein complex. Additionally, every residual contact in protein–protein interactions could be either favorable or unfavorable regarding the binding and affinity points of view^40,41^. Through MD simulations, the dynamicity of residue interactions is usually difficult to be delicately analyzed^36^. However, a recently developed tool, named gRINN method, has been introduced to generate and extract residue by residue behavior of the biomolecules during MD simulation. Using gRINN tool, the interaction energies, the significant residues and the correlations of interaction energies are obtainable^34^. The sum of total interaction energies in all residue-residue interactions was shown in each peptide_MDM2/X complexes (Fig. 3). Accordingly, as shown in Fig. 3A, the total interaction energies in pDIm1 (− 84.15 kcal/mol), pDIm2 (− 51.6 kcal/mol) and pDIdm (− 75.25 kcal/mol) decreased compared to that in pDI (− 95.89 kcal/mol) in complex with MDM2. However, the total interaction energy of pDIdm_MDMX was remarkably higher (− 87.5 kcal/mol) compared to the pDI_MDMX complex (− 32.65 kcal/mol) which made pDIdm peptide a potent dual inhibitor of MDM2/X. On the other hand, although the sum of interaction energies in pDIQ_MDM2 (− 85.7 kcal/mol) was comparable with pDI_MDM2 (− 95.89 kcal/mol), the level of interaction energies of pDIQ_MDMX (− 27.4 kcal/mol) was even lower than that of pDI_MDMX (− 32.65 kcal/mol). The interaction energy of pDIQm2 (− 23.4 kcal/mol) was lower in complex with MDM2 and was not different from that of MDMX (23.4 kcal/mol) compared to the pDIQ_MDM2/X interaction energies. In contrast, pDIQm1_MDM2 with total interaction energies of − 85.35 kcal/mol preserved the level of interaction energies of pDIQ_MDM2. However, its interaction energy quantity with MDMX was unremarkably higher (− 43.2 kcal/mole) compared to the pDIQ_MDMX complex. Overall, the top potent peptides against MDM2 in terms of the sum of residue-residue interaction energies were pDI (− 95.89 kcal/mol), PMI (− 92.6 kcal/mol), pDIQ (− 85.7 kcal/mol), pDIQm1 (− 85.35 kcal/mol) and pDIm1 (− 84.15 kcal/mol). However, regarding dual MDM2/X inhibition, the main potent peptide was pDIdm with − 75.25 kcal/mol and − 87.5 kcal/mol sums of interaction energies to MDM2 and MDMX, respectively.

To get a better view of the type of intermolecular forces playing roles in the row of p53-based peptides bound to MDM2/X, the sum of interaction energies were illustrated in the Fig. 3B, C. As shown in Fig. 3B, electrostatic forces played an important role in the experimentally modified p53 sequences such as pDI, pDIQ, and PMI. The sum of electrostatic interaction energies of the p53_MDM2 complex increased from − 10.955 kcal/mol to − 64.165 kcal/mol, − 53.56 kcal/mol and − 65.35 kcal/mol in pDI_MDM2, pDIQ_MDM2, and PMI_MDM2 complexes, respectively. Similarly, the mutant pDI and pDIQ peptides showed strong electrostatic interaction energies with MDM2. Exceptions were pDIm2 and pDIQm1 with the lower electrostatic type of interaction energies. On the other hand, as shown in Fig. 3C, van der Waals forces had a higher level in the modified p53_MDM2 complexes compared to the native p53_MDM2. However, , van der Waals energy values were not much different among the peptides in MDM2 complexes. As shown in Fig. 3B, C, regarding the type of interaction energies with MDMX, there was a similar level of electrostatic and van der Waals interaction energies (with a slight favor for electrostatic bonds). The only exception was pDIdm which showed the electrostatic energy of − 63.2 kcal/mol for MDMX. This was possibly the reason for the its best binding energy to MDMX (see the previous section) and the highest residue-residue sum of interaction energies.

**Figure 4:**
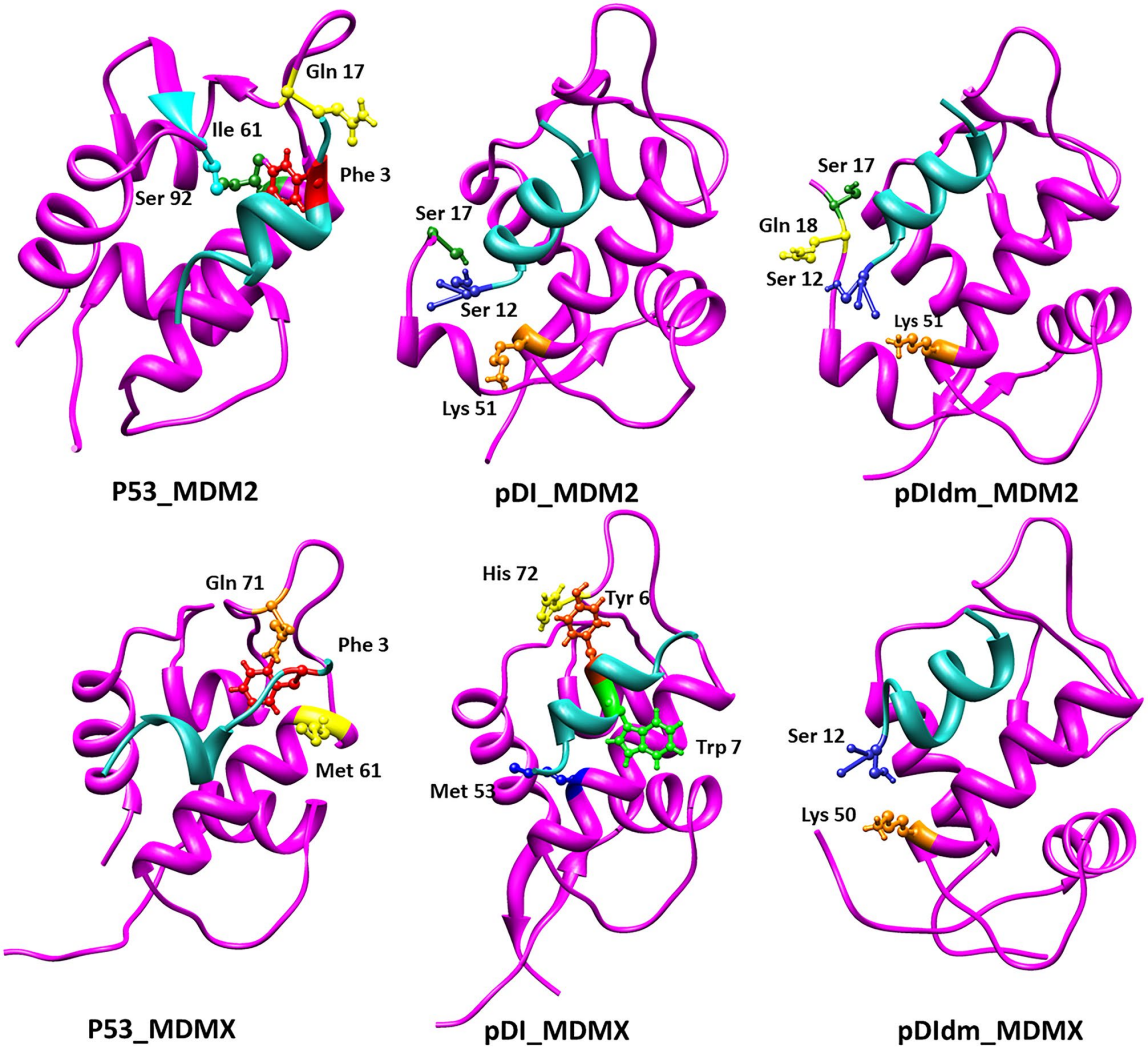
200 ns snapshots of MD simulations for p53, pDI, and pDIdm in complex with MDM2 and MDMX. It illustrates the critical residues in the interactions of the peptides_MDM2/X complexes. Data are generated by gRINN tool and images have been developed using Chimera software.

To show the important and specific residue-residue interactions in each peptide_MDM2/X structure, top interactions were illustrated in Fig. 4, Tables 2 and 3. Accordingly, Phe3, as one of the three main residues of p53_MDM2/X binding site had the most top interaction energies with MDM2 residues of Gln72, Ile61, Val93, and Gly58. In the pDI_MDM2, the strong interactions were between Ser12 of pDI and Lys51 and Ser17 of MDM2. Ser12 of pDIm1, pDIm2, and pDIdm had also the highest interaction energies with Lys51, Ser17, and Gln18 residues of MDM2, respectively. The Ser12 interaction energy remained at the highest level in the pDIQ and pDIQm1 in complex with MDM2. The pDIQm2 peptide with no Ser12 showed the higher interaction energy in phe3 residue which might have been an important reason for total low binding energy in the pDIQm2_MDM2 complex (see the previous section). In the PMI_MDM2 structure, Ser11 indicated a strong interaction with Lys51 of MDM2. Regarding MDMX complexes (Table 3), phe3 of p53 had a low level of interaction energy compared to that of p53_MDM2 complex. Modifying the p53 sequence, Tyr 6 and Trp7 of pDI with His72 and Met53 of MDMX showed the highest interaction energies in the pDI_MDMX complex. This was also the case in the pDIm1 and pDIm2 in complex with MDMX. However, in the pDIdm, the highest interaction energy was Ser12 with Lys50 of MDMX. On the other hand, Trp7 played an important role in the pDIQ and PMI interactions with MDMX. However, Glu4 and Leu11 were the critical residues of pDIQm1_MDMX and pDIQm2_MDMX complexes, respectively. The results were also depicted in Fig. 4 highlighting the change of critical interacted residues during the modification of the peptide sequences from p53 to pDIdm. Given the fact that pDIdm have shown better results compared to other peptides, it can be implied that Ser12 plays a most critical role in dual inhibition of MDM2/X proteins.

**Table 2:** Top 10 residues-residue interactions in peptides_MDM2 complexes with the highest interaction energy extracted from gRINN tool. The left residue is relevant to the peptide sequence and the right one to the binding site of MDM2. If applicable, the FoldX mutations will be shown in **bold** letters.

pDI-MDM2	Ave total E (kcal/mol)	pDIm1-MDM2	Ave total E (kcal/mol)	pDIm2-MDM2	Ave total E (kcal/mol)	pDIdm-MDM2	Ave total E (kcal/mol)
SER12-LYS51	− 21.35	SER12-LYS51	− 19.03	SER12-SER17	− 9.43	SER12-GLN18	− 8.22
SER12-SER17	− 13.63	SER12-SER17	− 15.21	PHE3-GLN72	− 6.29	PHE3-GLN72	− 5.51
THR11-LYS51	− 9.02	PHE3-GLN72	− 5.59	PHE3-TYR67	− 2.98	LEU10-ILE19	− 5.07
PHE3-GLN72	− 5.99	TRP7-LEU54	− 3.99	TYR6-HIS73	− 2.90	TRP7-LEU54	− 4.77
TRP7-PHE55	− 3.89	THR11-LYS51	− 3.85	TYR6-GLN72	− 2.73	GLN9-LYS94	− 3.02
TRP7-MET62	− 3.13	PHE3-TYR67	− 2.70	THR2-GLN72	− 2.67	GLN9-GLN18	− 2.98
TYR6-HIS73	− 2.94	TYR6-HIS73	− 2.58	TRP7-LEU54	− 2.52	**TRP4-MET62**	− 2.77
GLN9-SER17	− 2.94	THR2-GLN72	− 2.40	LEU10-SER17	− 1.89	PHE3-TYR67	− 2.67
LEU10-SER17	− 2.89	TYR6-GLN72	− 2.26	PHE3-MET62	− 1.72	SER12-PRO20	− 2.60
GLN9-HIS96	− 2.73	GLN9-SER17	− 2.16	**MET11-LEU54**	− 1.61	THR2-GLN72	− 2.39

pDIQ-MDM2	Ave total E (kcal/mol)	pDIQm1-MDM2	Ave total E (kcal/mol)	pDIQm2-MDM2	Ave total E (kcal/mol)		
SER12-ALYS51	− 29.41	SER12-ALYS51	− 25.49	PHE3-GLN72	− 6.61		
TRP6-AHIS96	− 6.75	SER12-AHIS96	− 10.15	PHE3-MET62	− 2.33		
TRP7-LEU54	− 6.19	LEU11-LYS51	− 6.05	THR2-GLN72	− 2.33		
PHE3-GLN72	− 5.21	PHE3-GLN72	− 5.93	TRP6-HIS73	− 1.96		
LEU11-LYS51	− 4.85	TRP7-PHE55	− 5.29	TRP6-GLN72	− 1.62		
THR2-GLN72	− 2.37	GLN9-HIS96	− 3.62	LEU11-LEU54	− 1.45		
LEU10-LYS51	− 2.34	TRP7-LEU54	− 3.06	PHE3-ILE61	− 1.28		
LEU10-LEU54	− 1.95	LEU10-HIS96	− 2.48	LEU11-PHE55	− 1.25		
PHE3-TYR67	− 1.87	LEU10-LEU54	− 2.36	PHE3-VAL93	− 1.04		
TRP7-PHE55	− 1.69	PHE3-MET62	− 2.21	SER12-LEU54	− 0.77		

P53-MDM2	Ave total E (kcal/mol)	PMI-MDM2	Ave total E (kcal/mol)				
PHE3-GLN72	− 6.87	SER11-ALYS51	− 25.93				
PRO11-LEU54	− 2.96	SER11-AHIS96	− 8.85				
THR2-GLN72	− 2.78	PHE3-AGLN72	− 5.87				
LEU10-LEU54	− 2.63	LEU10-ALYS51	− 5.41				
PRO11-PHE55	− 2.23	TRP7-APHE55	− 4.09				
SER4-GLN72	− 1.53	TRP7-AMET62	− 3.81				
PHE3-ILE61	− 1.18	TRP7-ALYS51	− 3.70				
PHE3-VAL93	− 1.02	LEU10-ALEU54	− 2.31				
PHE3-GLY58	− 1.01	TRP7-AGLY58	− 2.28				
LEU10-VAL93	− 1.00	SER11-ALEU54	− 2.27

**Table 3:** Top 10 residues-residue interactions in peptides_MDMX complexes with the highest interaction energy extracted from gRINN tool. The left residue is related to the peptide sequence and the right one to the binding site of MDMX. If applicable, the FoldX mutations will be shown in **bold** letters.

pDI-MDMX	Ave total E (kcal/mol)	pDIm1-MDMX	Ave total E (kcal/mol)	pDIm2-MDMX	Ave total E (kcal/mol)	pDIdm-MDMX	Ave total E (kcal/mol)
TYR6-HIS72	− 4.41	TYR6-HIS72	− 5.40	TRP7-HIS54	− 7.67	SER12-LYS50	− 26.58
TRP7-MET53	− 3.12	TRP7-MET53	− 4.89	PHE3-GLN71	− 5.03	ALA8-LYS50	− 9.51
PHE3-GLN71	− 2.96	PHE3-GLN71	− 3.43	LEU10-MET53	− 2.98	TRP7-MET53	− 5.96
TYR6-GLN71	− 2.56	PHE3-MET61	− 2.69	TYR6-HIS72	− 2.83	GLN9-LYS50	− 4.78
PHE3-TYR66	− 2.55	PHE3-TYR66	− 2.43	PHE3-MET61	− 2.80	**MET11-LYS50**	− 4.13
TRP7-ILE60	− 1.84	TYR6-GLN71	− 2.33	THR2-GLN71	− 2.37	LEU1-GLN71	− 3.93
TRP7-MET61	− 1.61	TRP7-LEU56	− 1.77	TRP7-MET61	− 2.12	SER12-MET53	− 3.55
PHE3-ILE60	− 1.54	PHE3-GLY57	− 1.75	TYR6-GLN71	− 1.82	PHE3-MET61	− 2.92
PHE3-MET61	− 1.52	THR2-GLN71	− 1.70	TRP7-MET53	− 1.24	**MET11-MET53**	− 2.48
THR2-HIS72	− 1.44	LEU10-PRO95	− 1.61	TRP7-GLY57	− 1.09	PHE3-TYR66	− 2.10

pDIQ-MDMX	Ave total E (kcal/mol)	pDIQm1-MDMX	Ave total E (kcal/mol)	pDIQm2-MDMX	Ave total E (kcal/mol)		
TRP7-MET53	− 6.14	GLU4-HIS54	− 9.18	LEU11-MET53	− 2.25		
PHE3-GLN71	− 3.21	TRP7-MET53	− 4.77	TRP7-MET53	− 2.12		
PHE3-MET61	− 2.24	TRP6-GLN71	− 3.41	PHE3-GLN71	− 2.07		
TRP7-VAL92	− 2.13	PHE3-GLN71	− 3.26	TRP7-HIS54	− 1.97		
LEU11-MET53	− 2.08	PHE3-TYR66	− 2.40	PHE3-MET61	− 1.92		
PHE3-GLY57	− 1.81	LEU11-MET53	− 2.19	TRP7-VAL92	− 1.62		
TRP7-LEU56	− 1.32	TRP7-GLN71	− 1.63	TRP7-ILE60	− 1.54		
TRP7-ILE60	− 1.23	TRP7-ILE60	− 1.52	LEU11-HIS54	− 0.96		
PHE3-ILE60	− 1.21	TRP7-HIS54	− 1.38	PHE3-TYR66	− 0.86		
TRP7-GLY57	− 0.86	GLU4-GLY57	− 1.36	THR2-GLN71	− 0.85		

P53-MDMX	Ave total E (kcal/mol)	PMI-MDMX	Ave total E (kcal/mol)				
PHE3-GLN71	− 3.19	TRP7-HIS54	− 8.41				
PHE3-MET61	− 2.59	PRO12-HIS54	− 6.17				
THR2-GLN71	− 1.92	PHE3-GLN71	− 6.04				
PHE3-ILE60	− 1.90	SER11-HIS54	− 4.81				
LEU10-MET53	− 1.16	LEU10-MET53	− 3.16				
LEU10-VAL92	− 0.95	TYR6-HIS72	− 2.56				
THR2-MET61	− 0.89	PHE3-MET61	− 2.30				
PHE3-GLY57	− 0.89	TRP7-MET61	− 2.14				
PHE3-HIS72	− 0.81	TYR6-GLN71	− 1.99				
PHE3-VAL92	− 0.66	SER2-GLN71	− 1.89				

**Figure 5:**
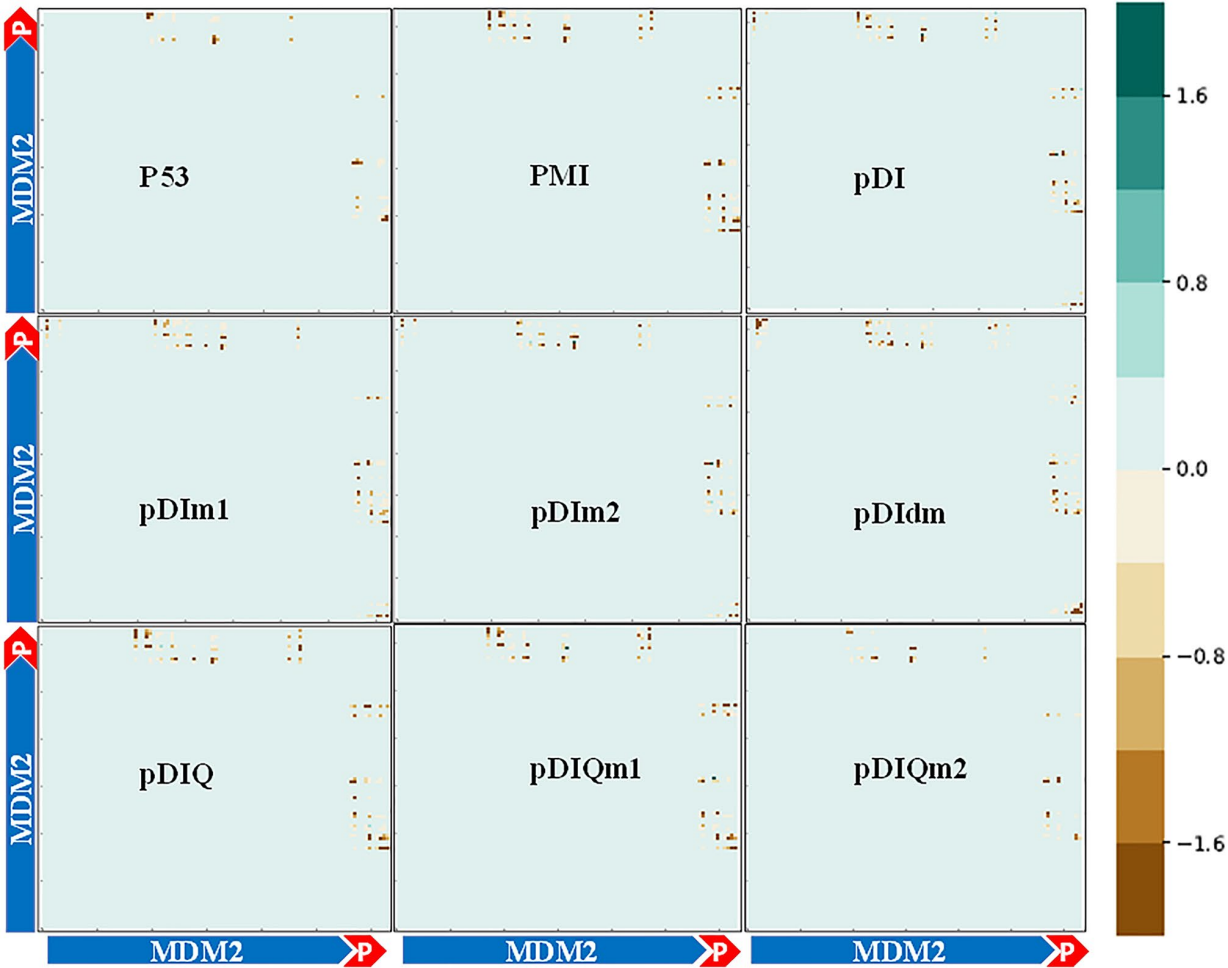
Interaction energy matrix that displays the average interaction energies between residue pairs of two chains in the studied peptides of p53, pDI, pDIm1, pDIm2, pDIdm, pDIQ, pDIQm1, pDIQm2 and PMI in complex with MDM2. According to the color range and the number of dots, the number of interactions, and the strongest bindings are shown.

**Figure 6:**
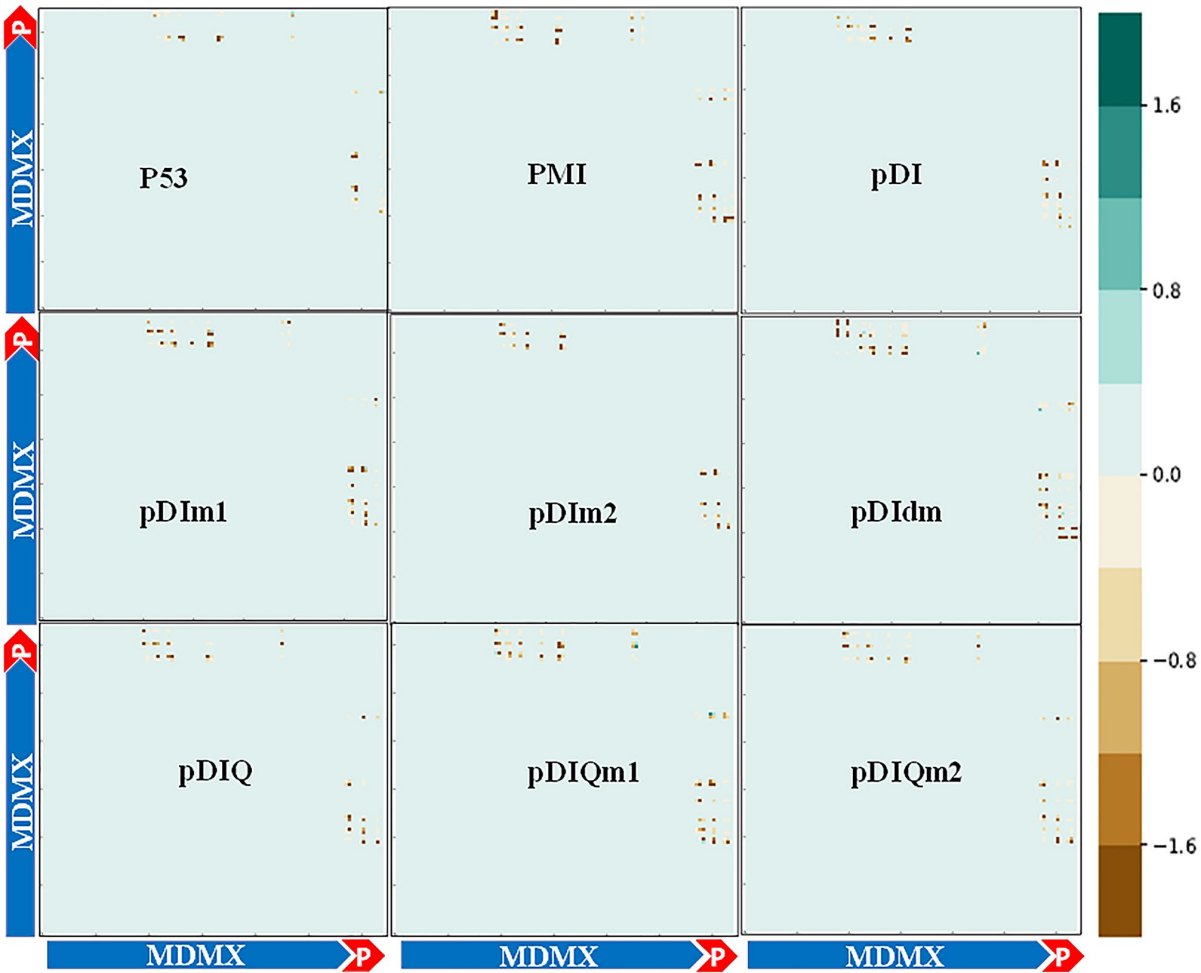
Interaction Energy Matrix that displays the average interaction energies between residue pairs of two chains in the studied peptides of p53, pDI, pDIm1, pDIm2, pDIdm, pDIQ, pDIQm1, pDIQm2 and PMI in complex with MDMX. According to the color range and the number of dots, the number of interactions, and the strongest bindings are shown.

The correlation of significant residues based on their location and interaction energies generated from the gRINN tool was shown in Figs. 5 and 6 for MDM2 and MDMX complexes, respectively. The dots in each graph have a color range of green to brown indicating the lower to higher residue-residue interaction energy, respectively. As shown in Fig. 5, the accumulation of interacted residues was higher in peptide_MDM2 regions. The intensity of brown dots in pDIm1, pDIm2, and pDIdm was remarkably higher compared to pDI bound to MDM2. On the other hand, in the pDIQm1_MDM2, while the dots accumulation was similar to pDIQ_MDM2, the level of green intensity was higher as well. However, the pDIQm2_MDM2 showed a decreased number of dots as well as higher brown intensity compared to the pDIQ_MDM2. Figure 6 shows the residue-residue correlation in peptides_MDMX interactions. The pDIdm_MDMX graph indicated a highest mass of residue-residue dots with higher interaction energies which is in accordance with previous results.

Historically, several anti-cancer peptides based on the p53_MDM2/X binding site have been developed mostly by using the phage display method. Some important ones include 12/1^16^, PMI^17^ and pDI^18^. The common limitation in all of the lead peptides was the lack of suitable affinity for MDMX. pDI, as one of the promising lead anti-MDM2/X peptides, was numerously applied for kinetics and dynamics improvements. In one of the first studies, in order to increase the affinity of the pDI, a structure–activity study was utilized by Phan et al.^23^. In this study, the MDM2/X IC_50_s of different mutant sequences of pDI were obtained and compared with p53, PMI and pDI. Ultimately, the pDIQ with the sequence of ET**F**EHW**W**SQ**L**LS was introduced as the most efficient dual inhibitory peptide with IC_50_ of 8 and 110 nM for MDM2 and MDMX, respectively (Table 1). Moreover, one of the clinically promising peptides, ALRN-6924, was originally optimized based on pDI sequence.

On the other hand, the suitable amount of experimental data regarding MDM2/X inhibitors over the last two decades has enabled scientists to feasibly use such data for the validation of theoretical methods. In one of the last attempts, Diller et al*.* described “CMDInventus” as a peptide design platform and a potent method to design inhibitory stapled peptides for MDM2/X^42^. Stapled peptides are usually designed to overcome the limitations of native peptides such as short plasma half-life. However, one of the promising approaches to design an efficient stapled peptide is first to achieve a native peptide sequence (a lead peptide) as a robust inhibitor of the target protein^43^. Afterward, synthetic modifications on the candidate peptides are likely to result in a more stable peptide-drug candidate. In the current study, it was attempted to study the p53-based lead peptides structurally in a way to guide the scientists to obtain more effective MDM2/X inhibitory peptides. In this path, the single or double mutations screening of known peptides was carried out. Mutant pDI and pDIQ, as well as, control peptides including p53, pDI, pDIQ, and PMI, went through MD simulations and corresponding analyses. Subsequently, the umbrella sampling method confirmed the experimental effectiveness pattern of the control peptides (Table 1). As a result, the method was confirmed as a suitable predicting approach for new developed peptides. In the literature, the PMI is the best native dual lead peptide with *Kd* of 3.3 and 8.9 nM for MDM2 and MDMX, respectively^17^. According to Table 1, ΔG_binding_ for PMI was calculated as 25 and 19 kcal/mol for MDM2 and MDMX, respectively, which is in high agreement with the experimental data. This is also true about pDI and pDIQ. Therefore, the theoretically designed mutant peptides, especially, pDIm1, PDIm2, and pDIdm, indicated promising ΔG_binding_ in dual inhibition of MDM2/X proteins. Another key factor in suitable inhibition of MDM2/X proteins is the conformation stability of the peptides. As shown in Fig. 4, 200 ns snapshots indicated no helical conservation of p53 peptide when they are bound to MDM2/X compared to other peptides. This is strongly relevant to p53 C-terminal residues specifically Pro11. On the other hand, in the modified peptides, mutations such as Pro11Met enhanced the conformation stability in both MDM2/X complexes.

Other follow-up analyses provided valid atomic-scale views of the peptide_MDM2/X interactions and their effectiveness. Accordingly, the results highlighted the importance of electrostatic forces during the interaction of peptides with MDM2/X. Besides, our data showed that Ser12 of modified peptides plays a key role in the effective MDM2/X dual inhibition. Therefore, while the last residue does not protect the helicity directly, it changes the interaction pattern in a way that the stability and affinity of the peptides increase. Moreover, Tyr6 and Trp7 are critical residues in the interaction of peptides with MDMX. These data are in agreement with those of the study that developed ATSP-7041 peptide. It shows additional Tyr6 interaction with MDMX binding pocket is an assistant to increase peptides_MDMX affinity^22^. Overall, the data confirmed the significance of the theoretical structure-based peptide development method for robust and rational dual anti-MDM2/X peptide design.

## Conclusion

MDM2 and MDMX are cooperative oncoproteins blocking the action of wild type p53 in several types of cancerous cells. Therefore, several MDM2/X inhibitory compounds have been developed over the last two decades to restore the normal proliferation of malignant cells. Some well-established lead peptides such as pDI and PMI have shown promising results in this regard. However, the peptides’ common limitation is the lack of dual inhibition of both MDM2/X proteins. As a result, in this study, it was attempted to develop a theoretical structure-based method fitting to the previous experimental data. This method was capable of analyzing the p53-based peptide_MDM2/X complexes in order to develop new native peptides with a higher affinity. In this path, previously tested peptides, including p53_MDM2/X binding site, pDI, pDIQ, and PMI as well as computationally screened mutants, named pDIm1, pDIm2, pDIQm1, and pDIQm2 in complex with MDM2/X were investigated using MD simulation. Detailed secondary structure analysis indicated that the peptides’ N-terminal residues contribute significantly to their helicity conservation when bound to MDM2 complexes. However, in the MDMX structures, the C-terminal residues of the peptides were more stable in the alpha-helix conformation. This contradiction makes it difficult to reach a potent, dual and stable peptide against MDM2/X proteins. Nevertheless, mutant pDI peptides, theoretically designed in this study, have shown to overcome this limitation. In addition, the ΔG_binding_ results obtained from the umbrella sampling method were in agreement with the potency of experimentally confirmed MDM2/X inhibitory peptides includingp53, pDI, pDIQ, and PMI peptides. Accordingly, the ΔG_binding_ of other screened mutant peptides, especially pDIdm, was reasonably promising. Moreover, having extracted residue by residue interaction energies, the critical residues of peptides_MDM2/X interactions were determined. Phe3 of p53 binding site was replaced with Ser12 in the modified peptides as critical interacting residue with MDM2. Lys51, Ser17, and Gln18 were the most critical residues of the interacting site of MDM2. Furthermore, since the Ser12 in pDIdm was conserved its interaction with MDMX, this case shows a high affinity to MDMX as well as MDM2. In conclusion, the result of this study highlighted the importance of atomic-scale and structure-based theoretical analyses to design potent peptides more rationally and efficiently. In conclusion, suggested rules as prediction structural markers of a promising anti-cancer p53-based peptide are including a) the presence of suitable C-terminal residues of p53-based peptides especially Glu9, Leu10, and Met11, b) the use of serine as the last residue, c) the appropriate ΔG_binding_ to MDM2/X, generated by the umbrella sampling method.

## Supplementary information

Supplementary file1 (PDF 650 kb)
