# Supplementary material for: Structure-based designing efficient peptides based on p53 binding site residues to disrupt p53-MDM2/X interaction
Source: Sci Rep. 2020 Jul 10;10:11449. doi: 10.1038/s41598-020-67510-8 (PMC7351717; doi:10.1038/s41598-020-67510-8)
Supplement: Supplementary file 1 — Supplementary file1 (PDF 650 kb) [file 41598_2020_67510_MOESM1_ESM.pdf]

Structure-based designing efficient peptides based on p53 binding site residues to disrupt p53-MDM2/X interaction

Nasim Rasafar[1], Abolfazl Barzegar[1*], Elnaz Mehdizadeh Aghdam[2,3*]

[1]*Research Institute of Bioscience and Biotechnology, University of Tabriz, Tabriz, Iran*

[2] *Molecular Medicine Research Center, Biomedicine Institute, Tabriz University of Medical Sciences, Tabriz, Iran*

[3]*Department of Pharmaceutical Biotechnology, Faculty of Pharmacy, Tabriz University of Medical Sciences, Tabriz, Iran*

* Co-corresponding authors:

Elnaz Mehdizadeh Aghdam
Email: mehdizadehe@tbzmed.ac.ir; aghdamelnaz@gmail.com
Phone number: 0098-41-33372250

Abolfazl Barzegar
Email: barzegar@tabrizu.ac.ir
Phone number: 0098-41-33393922

Table S1: The ΔE of each mutation in the pDI sequence when interacted with MDM2. The data are generated by the FoldX program. Each cell represents the difference of interaction energy of a single mutated pDI_MDM2 from the interaction energy of pDI_MDM2 in kcal/mol. The selected mutations are marked with **bold** digits.

pDI residues	Native Amino acids																			
	**A**	**C**	**D**	**E**	**F**	**G**	**H**	**I**	**K**	**L**	**M**	**N**	**P**	**Q**	**R**	**S**	**T**	**V**	**W**	**Y**
**L1**	-0.11	-0.04	-0.01	-0.05	-0.04	-0.15	0.00	0.00	-0.11	0.00	-0.23	-0.05	-0.02	-0.05	-0.13	-0.08	-0.07	-0.03	-0.13	-0.05
**T2**	0.28	-0.84	0.46	-0.85	-0.19	-0.39	-0.58	-1.26	-0.12	-0.96	-0.74	0.14	0.10	-0.62	-1.69	0.05	0.00	-0.64	-0.39	-2.39
**F3**	3.43	2.78	5.02	4.83	0.00	5.01	1.14	0.35	2.30	1.17	0.65	2.22	2.67	3.92	5.29	3.29	3.22	0.92	5.08	1.17
**E4**	-0.54	-0.25	0.05	-0.06	-1.38	-0.02	-0.58	-0.78	-0.32	-1.30	-1.44	-0.35	-0.70	-0.01	-0.90	0.12	-0.68	-0.84	**-1.97**	-0.98
**H5**	-0.03	-0.02	-0.09	-0.04	0.03	0.10	0.00	0.06	-0.04	-0.01	0.04	-0.01	-0.35	0.00	-0.02	-0.03	-0.04	0.03	0.01	-0.02
**Y6**	1.11	0.68	2.02	1.00	-0.42	1.80	0.38	0.10	0.65	-0.47	-0.07	1.24	1.66	1.14	1.63	1.77	0.80	-0.05	0.08	0.00
**W7**	5.86	5.55	6.64	6.51	5.27	6.17	4.40	4.82	5.17	4.32	3.31	6.24	7.78	5.79	5.76	6.19	5.97	5.45	-0.12	5.05
**A8**	0.00	-0.06	0.63	0.20	-0.11	-0.04	-0.12	0.38	-0.03	-0.02	-0.11	-0.07	0.32	-0.08	-0.21	-0.04	-0.04	-0.07	-0.15	0.05
**Q9**	-0.42	-0.11	-0.24	-0.23	0.11	-0.14	-0.12	-0.37	-0.12	-0.23	-0.16	-0.14	0.08	-0.01	0.14	-0.46	-0.02	-0.19	-0.09	0.05
**L10**	2.69	2.73	4.19	3.85	7.51	3.94	6.71	0.25	4.00	-0.02	-0.21	2.57	2.64	2.93	5.94	3.24	1.98	0.70	11.22	10.79
**T11**	0.43	-0.03	1.21	0.02	-0.80	0.89	-0.67	-1.51	-1.36	-1.92	**-1.58**	0.10	0.08	-0.22	0.38	-0.15	0.05	-0.54	0.15	-0.06
**S12**	0.56	0.62	-0.18	-0.53	-1.00	-0.34	-0.02	-0.57	-0.65	-0.99	-1.17	-0.07	0.85	-0.26	-0.53	0.00	0.02	-0.27	0.20	-0.03

Table S2: The ΔE of each mutation in the pDI sequence when interacted with MDMX. The data are generated by the FoldX program. Each cell represents the difference of interaction energy of a single mutated pDI_MDMX from the interaction energy of pDI_MDMX in kcal/mol. The selected mutations are marked with **bold** digits.

pDI residues	Native Amino acids																			
	**A**	**C**	**D**	**E**	**F**	**G**	**H**	**I**	**K**	**L**	**M**	**N**	**P**	**Q**	**R**	**S**	**T**	**V**	**W**	**Y**
**L1**	0.03	-0.01	0.02	0.27	-0.01	-0.16	-0.02	0.01	-0.08	0.00	-0.04	-0.03	0.06	0.04	-0.14	0.00	0.04	0.02	-0.09	-0.10
**T2**	-0.29	-0.39	0.24	-0.18	-0.30	0.08	-0.10	-0.01	0.00	-0.21	-0.24	-0.23	-0.06	-0.35	-0.24	-0.19	0.05	-0.04	-0.38	-0.36
**F3**	2.70	1.43	3.91	3.52	-0.04	4.68	1.96	1.47	2.74	0.58	-0.51	1.79	2.37	2.91	3.98	2.48	3.07	2.03	4.81	0.87
**E4**	-1.68	-1.68	0.32	0.00	-1.60	-1.65	-1.60	-2.51	-2.77	-1.66	-2.62	0.26	-1.36	0.01	-2.80	0.35	0.46	-1.77	**-2.90**	-1.64
**H5**	0.30	0.24	0.24	0.17	0.24	0.33	0.19	0.30	0.31	0.28	0.31	0.32	0.29	0.43	0.32	0.46	0.33	0.37	0.15	0.23
**Y6**	1.51	1.24	1.64	0.54	-0.10	2.39	1.02	0.31	0.97	-0.11	-0.16	1.56	1.82	1.25	1.73	2.02	1.43	0.10	-0.21	-0.01
**W7**	5.92	5.61	6.33	8.82	4.23	6.19	4.34	5.20	6.10	4.15	3.15	6.11	6.96	5.86	5.75	5.94	6.42	5.75	-0.02	4.35
**A8**	0.00	0.05	-0.23	-0.08	-0.10	-0.04	-0.03	0.05	0.23	0.08	0.09	-0.04	0.04	-0.01	0.09	0.01	0.04	0.04	0.02	-0.08
**Q9**	-0.10	-0.02	-0.14	-0.21	-0.05	-0.04	-0.11	0.17	-0.12	0.14	0.14	-0.02	0.31	0.00	-0.10	-0.18	-0.10	-0.01	-0.15	-0.09
**L10**	2.70	2.49	3.52	3.35	4.03	3.53	3.16	0.44	1.48	0.08	-0.77	2.09	3.88	2.63	1.43	3.28	2.34	2.14	4.02	11.94
**T11**	-0.01	-0.26	0.39	2.14	-0.17	0.18	0.15	-1.46	0.09	-1.41	**-2.02**	0.34	0.47	2.80	-0.13	0.14	0.00	-0.62	-0.26	-0.17
**S12**	-0.03	0.00	-0.08	-0.05	0.05	-0.12	0.02	0.01	0.08	0.00	0.02	0.00	0.08	0.08	0.17	0.00	0.01	0.07	0.01	0.01

Table S3: The ΔE of each mutation in the pDIQ sequence when interacted with MDM2. The data are generated by the FoldX program. Each cell represents the difference of interaction energy of a single mutated pDIQ_MDM2 from the interaction energy of pDIQ_MDM2 in kcal/mol. The selected mutations are marked with **bold** digits.

pDIQ residues	Native Amino acids																			
	A	C	D	E	F	G	H	I	K	L	M	N	P	Q	R	S	T	V	W	Y
E1	0.07	0.06	0.01	0.00	0.09	0.14	0.04	0.04	0.03	0.09	0.07	0.03	0.03	0.04	0.12	0.04	0.29	0.00	0.03	0.06
T2	-0.01	-0.11	0.09	-0.23	-0.23	0.14	**-1.28**	-0.10	-0.45	-0.49	-0.44	-1.23	-0.20	-0.39	-0.44	0.12	0.01	-0.14	-0.40	-0.50
F3	3.00	1.63	4.82	4.81	0.01	4.42	2.57	0.63	2.35	0.88	0.87	1.37	3.29	2.18	3.13	2.41	3.02	0.29	5.78	3.50
E4	-0.71	-0.06	0.10	0.00	-0.95	0.29	-0.31	-0.71	**-0.88**	-0.66	-0.36	-0.32	-0.29	-0.12	-0.90	0.13	-0.37	-0.30	-1.26	-0.38
H5	0.15	0.04	-0.08	-0.03	0.03	0.14	0.00	0.06	0.03	0.12	0.04	0.04	0.20	0.04	0.05	0.12	0.13	0.18	0.11	0.02
W6	1.37	1.27	2.25	1.59	-0.16	2.12	0.77	1.60	1.31	0.22	0.30	1.24	1.68	1.44	1.51	1.97	1.23	0.81	0.00	0.31
W7	5.27	4.87	6.37	6.30	4.05	5.41	4.63	4.99	4.44	4.08	2.90	5.27	7.75	5.27	4.94	5.31	6.05	4.93	0.16	4.96
S8	-0.01	0.06	-0.17	-0.06	-0.08	-0.12	0.05	0.02	0.11	0.19	-0.07	0.01	0.17	0.00	0.11	0.00	-0.04	0.02	-0.14	0.07
Q9	0.11	0.13	-0.10	-0.18	0.07	-0.04	0.18	0.12	0.08	-0.05	0.07	-0.01	0.03	0.00	0.15	-0.04	0.23	0.26	-0.18	0.08
L10	3.09	2.76	0.69	1.06	3.58	3.63	1.46	0.66	2.52	-0.21	-0.57	3.32	3.20	3.18	5.03	3.12	2.04	1.22	9.31	7.37
L11	2.15	1.45	2.02	1.70	1.17	1.99	2.04	0.57	0.80	-0.01	-0.34	2.07	2.58	1.73	1.36	2.20	0.41	1.51	2.31	1.07
S12	0.01	0.22	0.01	0.11	0.13	-0.04	0.34	0.39	0.24	0.34	0.25	0.07	0.31	0.26	0.36	0.00	0.06	0.32	0.30	0.28

Table S4: The ΔE of each mutation in the pDIQ sequence when interacted with MDMX. The data are generated by the FoldX program. Each cell represents the difference of interaction energy of a single mutated pDIQ_MDMX from the interaction energy of pDIQ_MDMX in kcal/mol. The selected mutations are marked with **bold** digits.

pDIQ residues	Native Amino acids																			
	**A**	**C**	**D**	**E**	**F**	**G**	**H**	**I**	**K**	**L**	**M**	**N**	**P**	**Q**	**R**	**S**	**T**	**V**	**W**	**Y**
**E1**	0.20	0.02	0.00	0.00	0.00	0.12	0.12	-0.09	0.01	-0.04	-0.09	-0.03	-0.06	0.01	-0.20	0.00	0.01	1.76	0.04	0.00
**T2**	-0.10	-0.06	-0.65	-0.39	-0.58	0.03	**-0.90**	-0.35	-0.54	-0.89	0.05	-0.07	-0.25	-0.35	-0.47	0.04	0.00	-0.13	-0.21	-0.49
**F3**	4.25	2.73	5.61	5.31	-0.01	5.41	1.57	1.66	2.54	1.65	0.83	3.01	4.09	3.73	6.19	3.85	4.41	2.07	5.22	0.45
**E4**	-0.30	-0.35	-0.06	-0.01	-0.77	0.07	-0.38	-0.90	**-0.84**	-0.98	-1.18	-0.19	-0.28	-0.44	-0.63	-0.03	-0.35	-0.56	**-0.59**	-0.71
**H5**	0.20	0.11	0.02	0.05	0.00	0.23	0.00	-0.06	0.10	-0.03	-0.03	0.09	0.17	0.18	0.08	0.14	0.23	0.23	-0.02	-0.01
**W6**	1.65	1.41	1.79	1.62	-0.24	2.53	0.51	0.79	1.09	-0.02	0.46	1.73	2.15	1.43	1.76	2.30	1.36	0.48	-0.10	-0.41
**W7**	6.06	5.73	6.59	6.84	3.33	6.50	4.32	5.33	5.71	4.85	3.50	6.27	7.64	6.28	5.81	6.42	6.11	5.68	-0.05	4.50
**S8**	-0.01	0.07	-0.06	-0.03	-0.33	0.00	-0.08	0.05	0.03	-0.06	0.04	-0.03	0.29	0.03	-0.21	-0.01	0.05	0.07	-0.77	-0.15
**Q9**	0.04	-0.04	-0.06	-0.06	0.05	0.03	0.17	0.05	0.07	0.04	-0.06	0.06	0.08	0.00	0.17	-0.01	0.00	0.08	0.04	-0.01
**L10**	2.44	2.28	1.84	2.63	5.26	3.45	1.97	1.71	1.14	-0.04	0.72	1.54	3.05	2.08	2.09	2.15	1.86	1.19	6.74	11.96
**L11**	2.31	2.13	1.26	1.37	-0.55	1.97	0.40	0.34	0.65	0.05	-0.71	1.53	1.75	1.27	1.07	2.34	2.37	2.27	0.07	-0.30
**S12**	0.11	0.13	0.02	0.03	0.12	0.01	0.15	0.23	0.29	0.15	0.12	0.13	0.57	0.12	0.45	0.00	0.05	0.19	0.14	0.17
